# K_v_1.1 channels mediate network excitability and feed-forward inhibition in local amygdala circuits

**DOI:** 10.1038/s41598-021-94633-3

**Published:** 2021-07-26

**Authors:** Samrat Thouta, Yiming Zhang, Esperanza Garcia, Terrance P. Snutch

**Affiliations:** 1 Michael Smith Laboratories, University of British Columbia, Vancouver, BC V6T 1Z4 Canada; 2 Djavad Mowafaghian Centre for Brain Health, University of British Columbia, Vancouver, BC Canada

**Keywords:** Biophysics, Neuroscience, Neurology

## Abstract

K_v_1.1 containing potassium channels play crucial roles towards dampening neuronal excitability. Mice lacking K_v_1.1 subunits (*Kcna1*^*−/−*^) display recurrent spontaneous seizures and often exhibit sudden unexpected death. Seizures in *Kcna1*^*−/−*^ mice resemble those in well-characterized models of temporal lobe epilepsy known to involve limbic brain regions and spontaneous seizures result in enhanced cFos expression and neuronal death in the amygdala. Yet, the functional alterations leading to amygdala hyperexcitability have not been identified. In this study, we used *Kcna1*^*−/−*^ mice to examine the contributions of K_v_1.1 subunits to excitability in neuronal subtypes from basolateral (BLA) and central lateral (CeL) amygdala known to exhibit distinct firing patterns. We also analyzed synaptic transmission properties in an amygdala local circuit predicted to be involved in epilepsy-related comorbidities. Our data implicate K_v_1.1 subunits in controlling spontaneous excitatory synaptic activity in BLA pyramidal neurons. In the CeL, K_v_1.1 loss enhances intrinsic excitability and impairs inhibitory synaptic transmission, notably resulting in dysfunction of feed-forward inhibition, a critical mechanism for controlling spike timing. Overall, we find inhibitory control of CeL interneurons is reduced in *Kcna1*^*−/−*^ mice suggesting that basal inhibitory network functioning is less able to prevent recurrent hyperexcitation related to seizures.

## Introduction

Epilepsy is a complex chronic neurological disease characterized by spontaneous and recurrent seizures resulting from abnormal electrical activity in the brain^1^. A growing body of evidence has identified and strongly implicated defects in several ion channel genes in the pathogenesis of epilepsy^2^. Voltage-gated K^+^ channel subunits K_v_1.1, encoded by the *KCNA1* gene, are broadly expressed in the mammalian brain, predominantly in the cortex, hippocampus, cerebellum and brainstem where they play important roles in dampening neuronal excitability^3^. K_v_1.1-containing channels mediate a number of functional roles associated with distinct underlying mechanisms and linked to their localization to discrete subcellular domains. K_v_1.1 subunits expressed in the soma affect the action potential waveform and firing patterns^4^; those located to the axonal compartment control the initiation and conduction of action potentials^5–7^, and at synaptic terminals they regulate neurotransmitter release^8,9^.

Loss-of-function missense mutations in the *KCNA1* gene are primarily associated with episodic ataxia type 1 (EA1), an autosomal dominant neurological disorder characterized by ataxia and muscle rippling^10^. Notably, a subset of patients with EA1 also exhibit seizures, further implicating mutations in the *KCNA1* gene in epilepsy^10–12^. In support of an epileptogenic role for *Kcna1* mutations, a *Kcna1* knock out mouse model (*Kcna1*^*−/−*^) exhibits phenotypes similar to patients with severe epilepsy as characterized by recurrent spontaneous seizures including myoclonic and generalized tonic–clonic seizures beginning at 3–4 weeks postnatally^13,14^. *Kcna1*^*−/−*^ mice also exhibit cardiorespiratory dysfunction and an increased risk of sudden unexpected death^15–18^. As such, *Kcna1*^*−/−*^ mice have been employed as a useful model for studying the cellular mechanisms underlying epileptic phenotypes.

Phenotypically, *Kcna1*^*−/−*^ mice resemble well-characterized kainate-and kindling-induced seizure models of temporal lobe epilepsy (TLE), suggesting *Kcna1* channel involvement in limbic brain circuits towards the generation and propagation of seizures^13,14,19,20^. EEG recordings of ictal and interictal activity from *Kcna1*^*−/−*^ mice showed that some seizure-like events recorded in the hippocampus precede the ictal episodes in the neocortex, and support the notion that epileptic seizures in this model originate in limbic structures^19^. Our current understanding of the mechanisms underlying epileptogenic and seizure activity in *Kcna1*^*−/−*^ mice is largely based on morphological and electrophysiological studies of hippocampal alterations^13,21,22^. Histological analysis of *Kcna1*^*−/−*^ mice experiencing seizures have provided evidence of hippocampal sclerosis, characterized by a selective neuronal cell loss in the CA1/CA3 region, degeneration of hilar interneurons, and sprouting of mossy fibers^19^. In vitro hippocampal slice recordings from *Kcna1*^*−/−*^ mice showing pathological high-frequency oscillations suggesting that network abnormalities contribute to seizure generation^22^. Given the role of the hippocampus in cognitive function e.g., learning and memory, deficits in hippocampal function are proposed to contribute to cognitive impairments associated with epilepsy^23^.

Studies have shown that the amygdala is a critical component for the onset and propagation of temporal lobe seizures^24,25^. Moreover, animal models of TLE show extensive neuropathology signs in the amygdala circuitry resembling those in human TLE^24^, including extensive loss of GABAergic neurons^26^. Additional evidence for a central role of the amygdala in the generation and propagation of seizure activity comes from kindling models of epilepsy in which the amygdala exhibits extreme susceptibility to electrically induced seizures^27^. Of the various amygdalar nuclei, the basolateral amygdala (BLA) both exhibits K_v_1.1 immunoreactivity ^28^ and has the highest propensity to generate seizures^29^. At the molecular and cellular levels, *Kcna1*^*−/−*^ mice experiencing status epilepticus display extensive neuronal cell loss and gliosis in the BLA region^19^ and spontaneous seizures resulting in significantly enhanced cFos expression in BLA neurons^20^. *Kcna1*^*−/−*^ mice also possess an enlarged amygdala correlated with seizure activity^30,31^. Overall however, disturbances in non-hippocampal temporal lobe network regions, and their functional contributions to epileptogenesis and behavioral impairments associated with seizures in *Kcna1*^*−/−*^ mice have remained relatively unexplored.

The central amygdala provides the main output of the amygdala complex, projecting to the hypothalamus and brainstem regions involved in the expression of emotional and autonomic responses^32,33^. Within the central amygdala, the central lateral subdivision (CeL) has received increasing interest due to its widespread function towards mediating fear responses^34^, anxiety^35,36^ and nociception^37,38^. Dysfunction of the amygdala has been linked to epilepsy-related mood disorders, contributing to the high comorbid occurrence of anxiety and depression in patients with epilepsy^39^. Moreover, it has been suggested that the amygdala is also involved in respiratory abnormalities associated with epilepsy^40^. Notably, *Kcna1*^*−/−*^ mice demonstrate respiratory failures including central apnea during seizures that can result in the phenomenon known as sudden unexpected death in epilepsy (SUDEP)^16–18^. A recent hypothesis proposes that spreading depolarization and subsequent brainstem dysfunction triggers respiratory failure in *Kcna1*^*−/−*^ mice^16^. Further, studies from both SUDEP patients^41–43^ and mice^44^ suggest that the amygdala, specifically the central amygdala, has a significant effect on seizure-induced apneas.

Here, we examined whether electrophysiologically distinct neuronal cell types from the CeL and BLA nuclei of *Kcna1*^*−/−*^ mice exhibit alterations in intrinsic excitability and synaptic activity. Since the BLA appears to be a key site for seizure initiation in TLE, we first examined the functional characteristics of BLA pyramidal neurons and found that the lack of K_v_1.1 subunits alters spontaneous synaptic activity in BLA pyramidal neurons with no changes in firing responses evoked by somatic stimulation. We also provide evidence for the loss of K_v_1.1 subunits towards enhancing intrinsic excitability and impairing feed-forward inhibition into CeL GABAergic neurons. Together, we predict that these changes in synaptic and circuit properties contribute to neuronal hyperexcitability in *Kcna1*^*−/−*^ mice and to promote seizure activity.

## Results

### Loss of K_v_1.1 subunits in BLA pyramidal neurons enhances excitatory synaptic activity but does not affect inhibitory synaptic activity or intrinsic excitability

**Figure 1:**
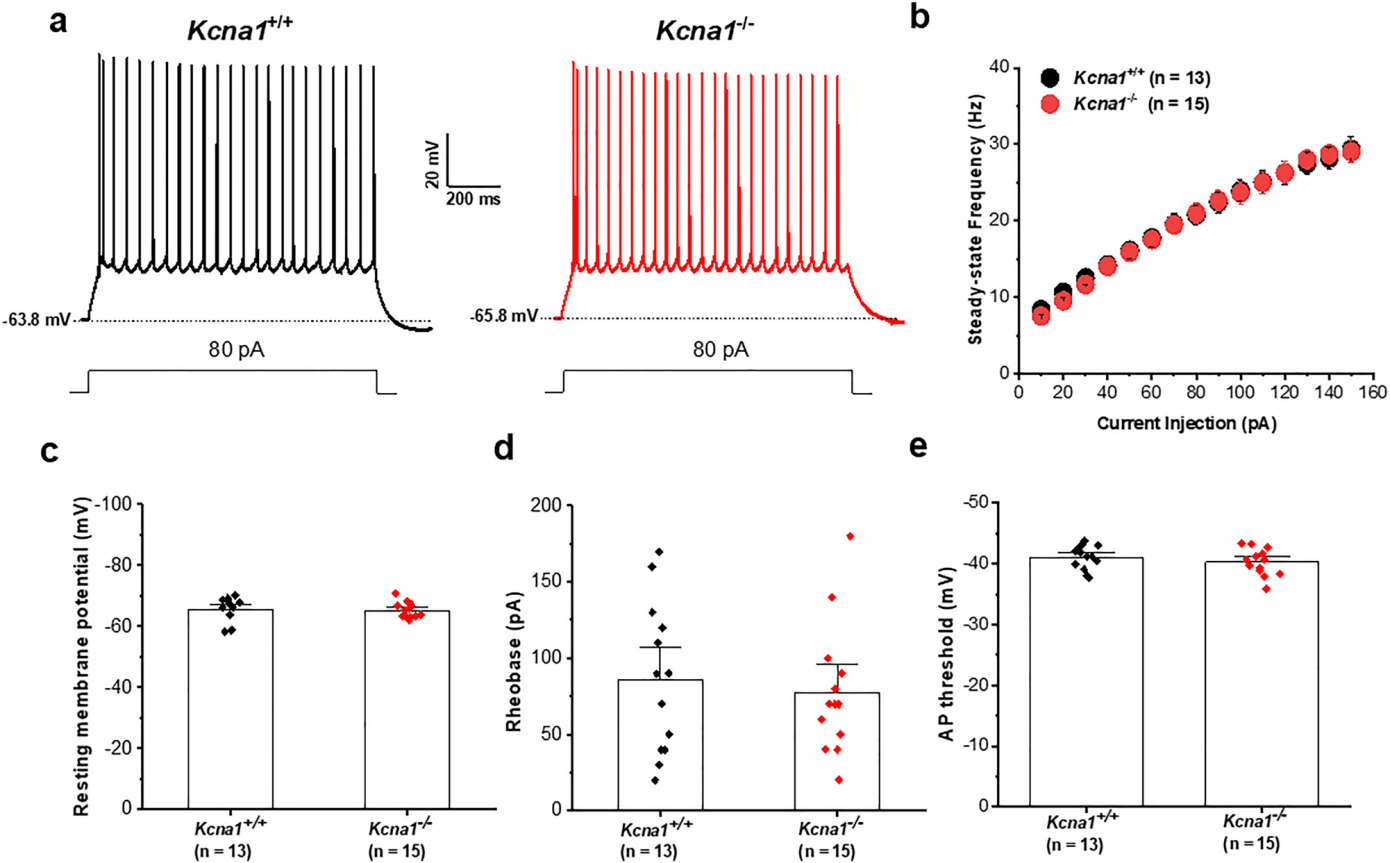
Lack of effect of K_v_1.1 deletion on the intrinsic excitability of BLA pyramidal neurons. **(a)** Representative current-clamp recordings of BLA pyramidal neurons from both *Kcna1*^+*/*+^ (black trace) and *Kcna1*^*−/−*^ (red trace) mice recorded in response to a 80-pA stimulus. Dotted lines indicate the resting membrane potential. **(b)** Mean current–frequency (*f*–I) plot showing firing frequency in response to current injections of increasing magnitude. There was no change in the firing frequency in BLA neurons from *Kcna1*^*−/−*^ compared to *Kcna1*^+*/*+^ mice. **(c–e)** Bar graphs show the average values of resting membrane potential (**c**), rheobase (**d**) AP threshold (**e**) and symbols represent the individual cell values. No significantly difference was obtained (*p* > 0.05). *Kcna1*^+*/*+^, n = 13 cells from 5 mice; *Kcna1*^*−/−*^, n = 15 cells from 4 mice.

**Figure 2:**
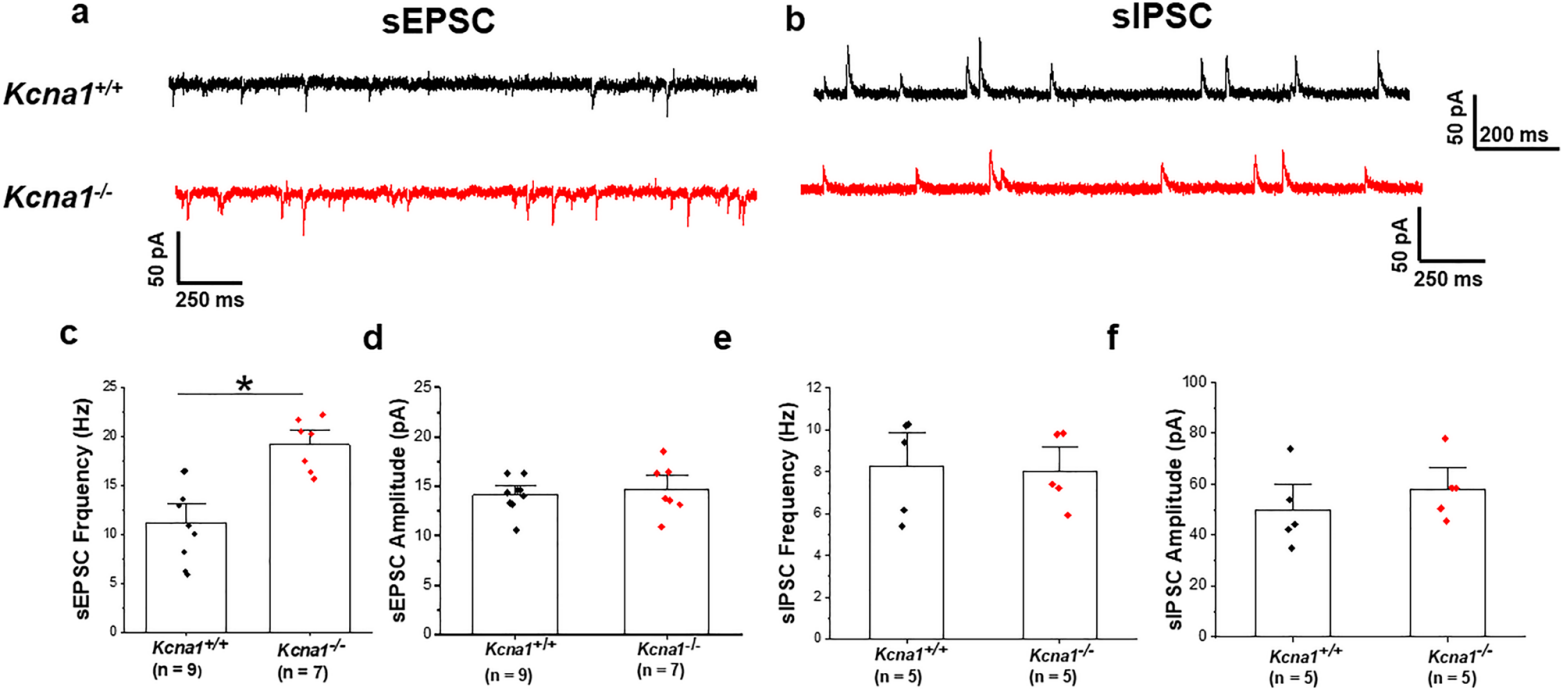
K_v_1.1 deletion enhances spontaneous postsynaptic excitatory activity in BLA pyramidal neurons. Representative sEPSCs (**a**) and sIPSCs (**b**) were recorded from BLA pyramidal neurons in *Kcna1*^+*/*+^ (black traces) and *Kcna1*^*−/−*^ (red traces) mice. For sEPSCs, cells were held at a holding potential of − 70 mV and were recorded in presence of PTX (100 µM). For sIPSCs, cells were held at a holding potential of 0 mV and were recorded in presence of CNQX (20 µM) and D-AP5 (50 µM). (**c,d**) Mean data of sEPSCs frequency (**c**) and amplitude (**d**), showing an increase in sEPSCs frequency and no difference in amplitude*. Kcna1*^+*/*+^, n = 9 cells from 5 mice; *Kcna1*^*−/−*^, n = 7 cells from 5 mice. (**e,f**) Mean data of sIPSCs frequency (**e**) and amplitude (**f**) showing no significant changes in sIPSCs between *Kcna1*^*−/−*^ and *Kcna1*^+*/*+^ mice. *Kcna1*^+*/*+^, n = 5 cells from 4 mice; *Kcna1*^*−/−*^, n = 5 cells from 4 mice. *p < 0.05*.*

Seizure related neuronal cell loss, gliosis and enhanced Fos immunostaining have been observed in the BLA of *Kcna1*^*−/−*^ mice^19,20^, however experimental evidence showing functional alterations associated to BLA in this animal model remain lacking. Employing whole-cell current clamp recordings of BLA pyramidal neurons from *Kcna1*^+*/*+^ and *Kcna1*^*−/−*^ mice, we initially examined whether the loss of K_v_1.1 subunits in BLA pyramidal neurons affects their intrinsic excitability and synaptic activity (Figs. 1 and 2). In response to incremental steps of current injection, BLA pyramidal neurons from both *Kcna1*^+*/*+^ and *Kcna1*^*−/−*^ mice exhibited similar action potential firing profiles with similar frequencies as shown in frequency-current (*f*-I) plots (Fig. 1b). We did not observe any difference between *Kcna1*^+*/*+^ and *Kcna1*^*−/−*^ mice in resting membrane potential (Fig. 1c), rheobase—the minimal current required to evoke action potential firing (Fig. 1d) or action potential threshold (Fig. 1e). Thus, loss of K_v_1.1 subunits does not appear to modify the intrinsic excitability of BLA pyramidal neurons. These data are consistent with previous results showing that kindling or repeated seizures in the amygdala do not alter membrane properties and intrinsic excitability of BLA pyramidal neurons^45,46^.

Over the last decades, there has been growing evidence that K_v_1.1 channel activity regulates neurotransmitter release, providing fine-tuning mechanisms to modulate synaptic strength^47,48^. Although studies have shown that presynaptic K_v_1.1-containing channels are localized in the BLA neurons^49^, their functional contributions to synaptic activity have not been reported.

We examined spontaneous excitatory (sEPSCs) and inhibitory (sIPSCs) postsynaptic currents in BLA pyramidal neurons in *Kcna1*^+*/*+^ and *Kcna1*^*−/−*^ mice (Fig. 2). Figure 2a shows an example of sEPSCs recorded in BLA neurons of *Kcna1*^+*/*+^ and *Kcna1*^*−/−*^ mice. The mean frequency of sEPSCs was significantly increased in *Kcna1*^*−/−*^ mice (18.8 ± 0.4 Hz, n = 7 cells from 5 mice) compared with *Kcna1*^+*/*+^ (12.8 ± 0.4 Hz, n = 9 cells from 5 mice, *p* = 0.0004; Fig. 2c). In contrast, the mean amplitude of sEPSCs showed no difference between *Kcna1*^+*/*+^ and *Kcna1*^*−/−*^ BLA pyramidal neurons (*Kcna1*^+*/*+^  = 14.7 ± 1.1 pA, n = 9 cells from 5 mice; *Kcna1*^*−/−*^ = 15.2 ± 1.1 pA, n = 7 cells from 5 mice *p* = 0.64; Fig. 2d). These data suggest that the increase in sEPSCs frequency observed in *Kcna1*^*−/−*^ BLA neurons is likely a result of an increased glutamate release probability at nerve terminals, consistent with previous reports of synaptic activity of BLA neurons from kindled rats^46^. We next recorded sIPSCs (Fig. 2b) and found no significant changes in either sIPSCs frequency or amplitude between *Kcna1*^+*/*+^ and *Kcna1*^*−/−*^ mice (Fig. 2e,f). The mean sIPSCs values were 8.2 ± 0.9 Hz and 49.7 ± 6.03 pA for *Kcna1*^+*/*+^ mice (n = 5 cells from 4 mice), and 8.03 ± 0.7 Hz and 58.1 ± 4.9 pA for *Kcna1*^*−/−*^ mice (n = 5 cells from 4 mice), respectively. To assess the net effect of altered spontaneous postsynaptic current (sPSC) activity onto *Kcna1*^*−/−*^ BLA pyramidal neurons, we calculated the sEPSCs/sIPSCs ratios of each measure (frequency and amplitude) for each cell and compared the ratio of the mean values between *Kcna1*^*−/−*^ and *Kcna1*^+*/*+^ mice. The mean ratio of frequency of sEPSCs to sIPSCs in *Kcna1*^*−/−*^ (2.4 ± 0.2, n = 5 cells from 4 mice) was significantly increased compared with *Kcna1*^+*/*+^ mice (1.1 ± 0.3, n = 5 cells from 4 mice, *p* = 0.003), indicating that the balance between excitation and inhibition in *Kcna1*^*−/−*^ mice is shifted toward excitation. The mean ratio of sEPSCs to sIPSCs amplitude was no different between *Kcna1*^+*/*+^ and *Kcna1*^*−/−*^ mice (*Kcna1*^+*/*+^  = 0.31 ± 0.04, n = 5 cells from 4 mice; *Kcna1*^*−/−*^ = 0.26 ± 0.02, n = 5 cells from 4 mice). Overall, there is an increase in excitatory events in BLA pyramidal neurons that may contribute to the seizure induced pathological changes in BLA of *Kcna1*^*−/−*^ mice. Of note, a previous report shows that presynaptic alterations of glutamate transmission in BLA neurons is associated with both increased anxiety and seizure generation^50^.

### K_v_1.1-containing channels regulate firing properties of late-spiking GABAergic interneurons in the central lateral amygdala

Loss of GABAergic interneurons and alterations in inhibitory activity has been demonstrated in the amygdala of epileptic animal models, suggesting that GABAergic neurons may play key roles in the generation and spread of seizures^26,51^ . Within the amygdala complex, the CeL consists almost exclusively of GABAergic interneurons that shape amygdala output and function as a relay station between the amygdala complex and other downstream targets^52–54^. K_v_1.1-containing channels play crucial roles in regulating near-threshold properties and repetitive firing of fast-spiking neocortical^6,55^ and deep cerebellar nuclear GABAergic neurons^56^ thus here we studied their contributions towards regulating excitability of CeL GABAergic interneurons.

**Figure 3:**
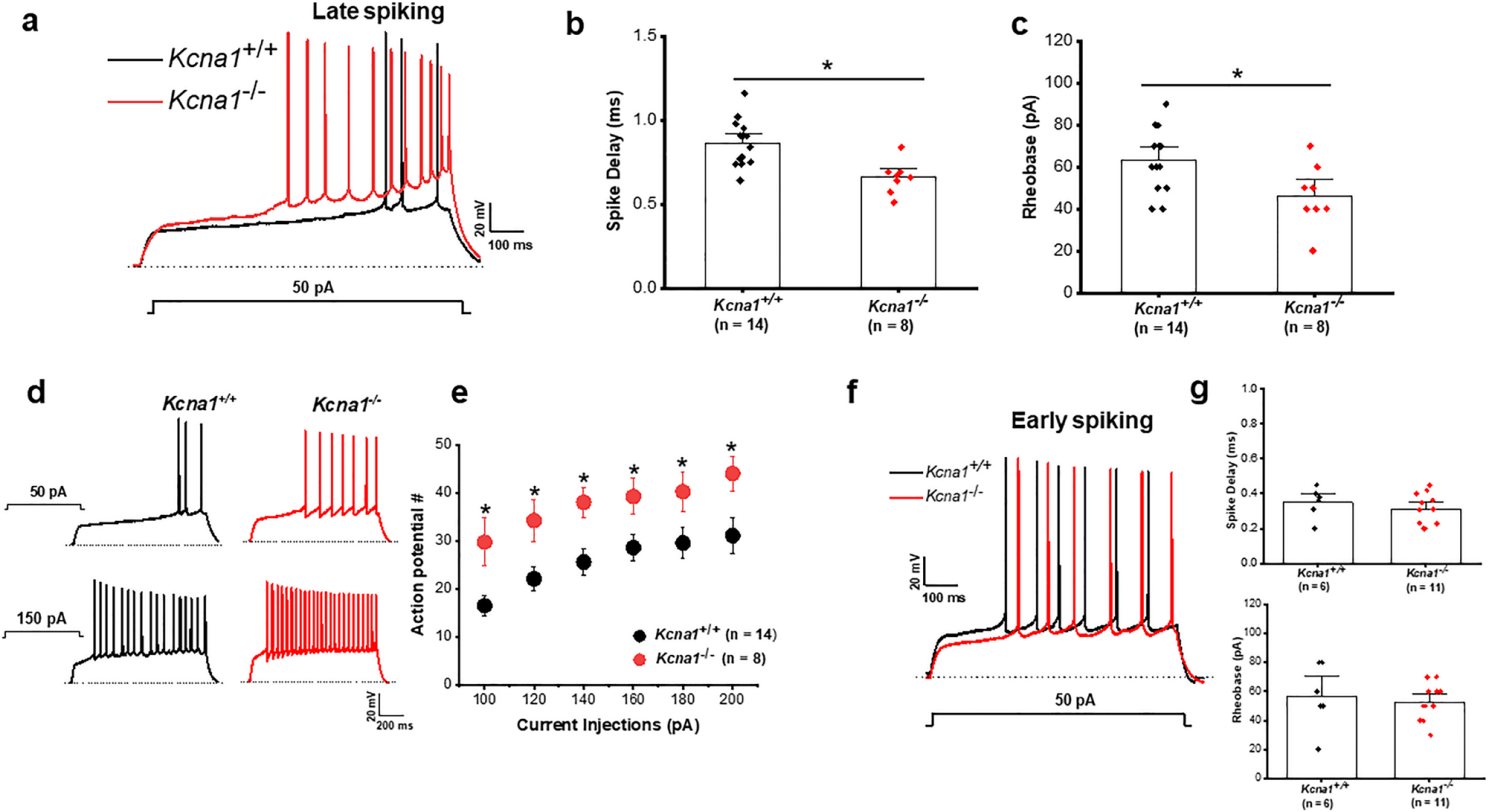
K_v_1.1-containing channels regulate intrinsic firing properties of late-spiking (LS) but not early spiking (ES) central lateral amygdala neurons. (**a**) CeL superimposed representative traces show AP firing of LS neurons from *Kcna1*^+*/*+^ (black trace) and *Kcna1*^*−/−*^ (red trace) recorded in response to a 50-pA stimulus. (**b,c**) Pooled data showing significant decrease in the spike delay (**b**) and rheobase (**c**) in CeL LS neurons of *Kcna1*^*−/−*^ compared to *Kcna1*^+*/*+^ mice. *Kcna1*^+*/*+^, n = 14 cells from 6 mice; *Kcna1*^*−/−*^, n = 8 cells from 7 mice. (**d**) Representative traces of repetitive firing elicited by depolarizing current injection of 50 (upper traces) and 150 pA (lower traces) in LS neurons from *Kcna1*^+*/*+^ (black traces) and *Kcna1*^*−/−*^ (red traces) mice. The traces illustrate the increase in the firing frequency of action potentials in both *Kcna1*^+*/*+^ and *Kcna1*^*−/−*^ mice in response to stimulus of higher magnitude. (**e**) The number of action potentials elicited by 1.2 s current injections was plotted against current injection to show the firing response of LS neurons as a function of the stimulus intensity. LS neurons from *Kcna1*^*−/−*^ mice showed increased AP firing compared to *Kcna1*^+*/*+^ mice suggesting that LS neurons of CeL from *Kcna1*^*−/−*^ possess increased intrinsic excitability. (**f**) Superimposed representative traces showing AP firing of ES neurons *Kcna1*^+*/*+^ (black trace) and *Kcna1*^*−/−*^ (red trace) mice recorded in response to a 50-pA stimulus. (**g**) In contrast to LS neurons, ES neurons showed no difference either in the spike delay (top) or rheobase (bottom) in *Kcna1*^*−/−*^ compared to *Kcna1*^+*/*+^ mice. *Kcna1*^+*/*+^, n = 6 cells from and 6 mice; *Kcna1*^*−/−*^, n = 11 cells from 7 mice. Dotted lines indicate the resting membrane potential. *p < 0.05.

Whole cell current-clamp recordings of CeL interneurons was used to compare firing properties between *Kcna1*^+*/*+^ and *Kcna1*^*−/−*^ mice. In response to depolarizing current injections, CeL neurons were shown to display two major firing patterns: late spiking (LS) and early spiking (ES), typical of that previously reported^52,54,57,58^. In control *Kcna1*^+*/*+^ mice, LS neurons exhibited a substantial delay before the onset of the first AP (~ 0.8 to 1 s; see Fig. 3a,b) while ES neurons displayed a shorter delay ~ 0.3 to 0.5 s (Fig. 3f,g, top panel). In contrast for LS CeL neurons from *Kcna1*^*−/−*^ mice, depolarizing current injection resulted in a rapid rise in membrane potential leading to an initial action potential after a short delay, as shown in the overlapped traces in Fig. 3a. Analysis of spike latency showed that in *Kcna1*^*−/−*^ mice, the spike latency to the first AP was significantly shorter (0.65 ± 0.04 ms, n = 8 cells from 7 animals; *p* = 0.001) compared to *Kcna1*^+*/*+^ mice. (0.86 ± 0.03 ms, n = 14 cells from 6 animals; Fig. 3b). Also, the rheobase was reduced in *Kcna1*^*−/−*^ LS cells (*Kcna1*^+*/*+^  = 70 ± 7.9 pA, n = 14 cells from 6 animals; *Kcna1*^*−/−*^ = 46.2 ± 4.6 pA, n = 8 cells from 7 animals; *p* = 0.04; Fig. 3c). These data suggest that K_v_1.1-containing channels are involved in determining the near-threshold behavior of CeL LS cells. Our data are consistent with findings in other brain regions such as neocortex ^6^ and dentate gyrus^59^ in which pharmacological blockade of K_v_1.1 altered spike latency and rheobase of LS cells. As shown in Fig. 3d, prolonged depolarizing current injections at suprathreshold voltages elicited repetitive firing in LS neurons from both *Kcna1*^+*/*+^ (black traces, left) and *Kcna1*^*−/−*^ (red traces, right); after a brief spike delay, LS cells fire at high frequencies with relatively little or no spike frequency adaptation. In both *Kcna1*^+*/*+^ and *Kcna1*^*−/−*^ neurons, the number of action potentials increased monotonically with stimulus strength; however, the LS neurons from *Kcna1*^*−/−*^ mice fired an increased number of APs during 1.2 s of current injections (*p* = 0.02; Fig. 3e). Interestingly, the effects of K_v_1.1 deletion on the excitability of CeL neurons was restricted to LS neurons as both intrinsic firing and passive membrane properties were indistinguishable in ES CeL neurons in both *Kcna1*^+*/*+^ and *Kcna1*^*−/−*^ mice (Fig. 3f), suggesting that the level of expression of K_v_1.1 might be higher in LS neurons. The mean values of spike latency and rheobase of ES neurons were 0.35 ± 0.03 ms and 56.6 ± 3.1 pA for *Kcna1*^+*/*+^ mice (n = 6 cells from 6 mice; Fig. 3g, top), and 0.31 ± 0.02 ms and 52.2 ± 3.8 pA for *Kcna1*^*−/−*^ mice (n = 11 cells from 7 mice; Fig. 3g, bottom), respectively. Together, these results show that loss of K_v_1.1 subunits dramatically enhances the intrinsic excitability of LS cells of the CeL, and which would be predicted to impact the output of the amygdala circuitry.

### Reduction of GABAergic inhibition in CeL neurons in *Kcna1*^*−/−*^ mice

**Figure 4:**
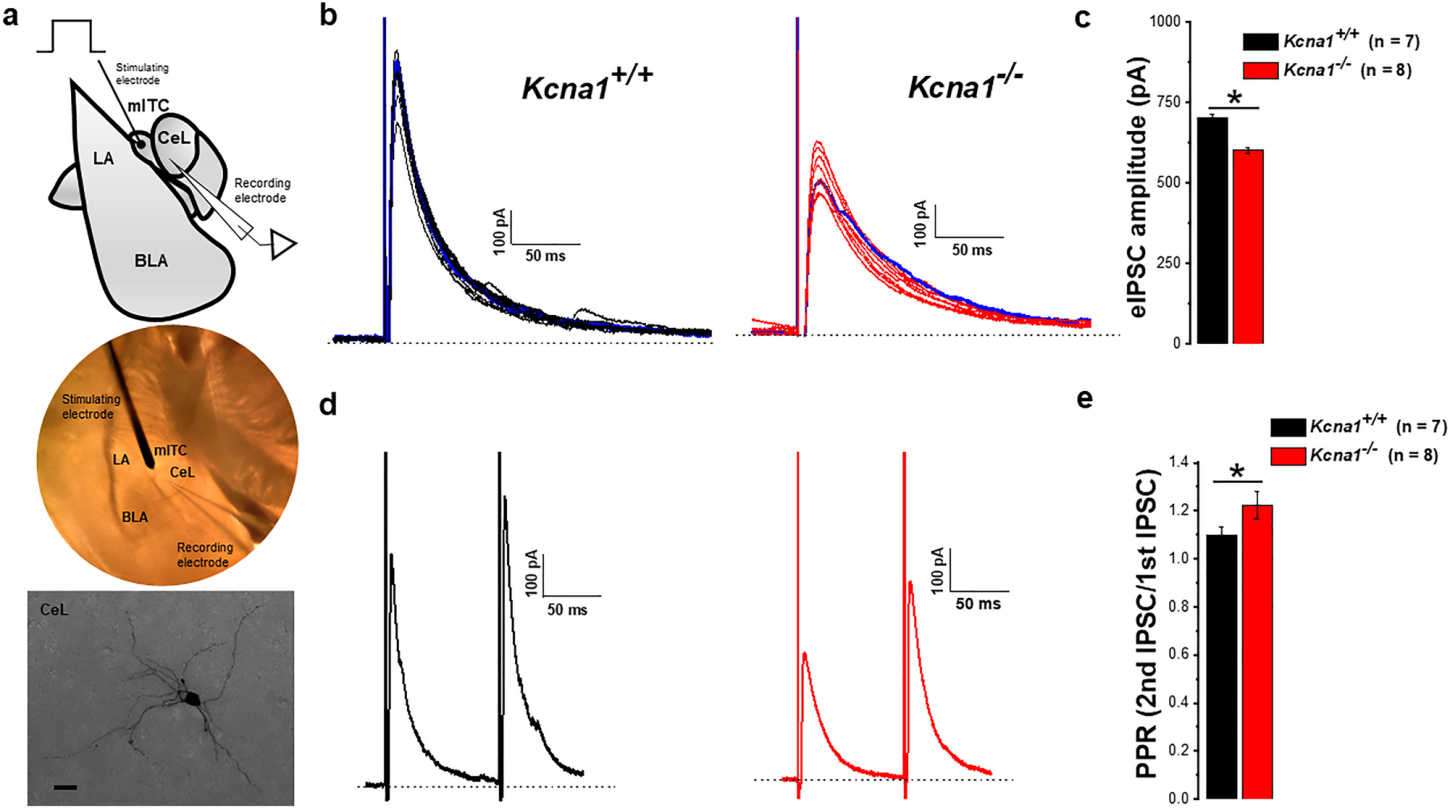
Inhibitory transmission and short-term plasticity at mITC-CeL GABAergic synapses are altered in *Kcna1*^*−/−*^ mice. (**a)** Schematic (top) and representative image of experimental preparation (*middle*) of a coronal section of amygdala complex. Note the stimulation electrode placed on the mITCs to evoke inhibitory postsynaptic currents (eIPSCs) in the CeL. Bottom: Image of a representative biocytin-filled recorded CeL neuron showing its typical medium-spiny morphology. Scale bar: 20 µm (**b) **Representative current traces (10 consecutive traces overlapped with average trace in blue) of eIPSCs from *Kcna1*^+*/*+^ (black traces) and *Kcna1*^*−/−*^ (red traces) mice recorded from a holding potential of 0 mV. (**c**) Mean values of eIPSCs amplitude calculated from the average responses obtained for each cell at a frequency of 0.1 Hz. The data show that the amplitude of eIPSCs at mITC-CeL synapses is significantly decreased in *Kcna1*^*−/−*^ compared to *Kcna1*^+*/*+^ mice, suggesting that basal synaptic strength is impaired at mITC-CeL synapse of *Kcna1*^*−/−*^*. Kcna1*^+*/*+^, n = 7 cells from 4 mice; *Kcna1*^*−/−*^, n = 8 cells from 6 mice. **(d**) Representative averaged trace obtained with a paired-pulse protocol (10 successive responses at 10 Hz) showing eIPSCs short-term facilitation in CeL neurons from *Kcna1*^+*/*+^ (black trace) and *Kcna1*^*−/−*^ (red trace) mice. A train of 10 pulses at 10 Hz was used (**e**) Mean values of pair-pulse ratio (PPR) evoked by pair stimuli (100 ms interstimulus interval) in mITC-CeL synapses. The PPR is increased in *Kcna1*^*−/−*^ compared to *Kcna1*^+*/*+^ mice. These data suggest that the release probability from presynaptic GABAergic terminal at mITC-CeL synapse is decreased in *Kcna1*^*−/−*^ mice. *Kcna1*^+*/*+^, n = 7 cells from 4 mice; *Kcna1*^*−/−*^, n = 8 cells from 6 mice. Dotted lines indicate the base line*.* LA, Lateral amygdala; BLA, Basolateral amygdala; CeL, Central lateral amygdala; and mITC, medial intercalated cells. The schematic (**a**, top panel) was made using curved line tool in PowerPoint and the image of biocytin filled recorded CeL neuron (**a**, bottom panel) was processed using the ImageJ-Fiji version 1.53j. *p <  0.05.

The CeL receives convergent excitatory inputs from the parabrachial nucleus in the brainstem and from the lateral amygdala (LA)^32,60,61^. The LA influences CeL function via direct glutamatergic projections and through indirect disynaptic connections involving intercalated cells (ITC) to generating feedforward inhibition to CeL neurons^53,62,63^. ITCs comprise distinct clusters of GABAergic neurons located along the lateral and medial borders of basolateral complex. Medial ITC neurons (mITCs) gate signaling from the LA into the CeL and have been shown to play a crucial role in fear extinction^64^. mITCs are thought to exert fine-tuning inhibitory control of CeL output pathways and here we investigated whether loss of K_v_1.1 subunits alters synaptic responses in CeL neurons elicited by the activation of direct projections from mITCs. We recorded evoked inhibitory post synaptic currents (eIPSCs) from CeL neurons at 0 mV in the presence of D-AP5 (50 µM), CNQX (20 µM) and CGP52432 (1 µM). Figure 4b shows representative eIPSC traces (10 consecutive traces superimposed with the average in blue) evoked at a frequency of 0.1 Hz from both *Kcna1*^+*/*+^ (black traces) and *Kcna1*^*−/−*^ mice (red traces). Compared to *Kcna1*^+*/*+^, the mean amplitude of eIPSCs was significantly reduced in *Kcna1*^*−/−*^ mice (*Kcna1*^+*/*+^  = 704.04 ± 9.2 pA, n = 7 cells from 4 mice; *Kcna1*^*−/−*^  = 602.2 ± 8.8 pA, n = 8 cells from 6 mice; *p* = 0.00001; Fig. 4c). These results suggest that basal synaptic strength at mITC-CeL synapses is attenuated in *Kcna1*^*−/−*^ mice.

To explore mechanisms underlying the observed changes in synaptic strength we next measured the paired-pulse ratio (PPR) as an index of short-term facilitation, a known form of plasticity at mITC-CeL synapses. Figure 4d show representative traces of eIPSCs evoked by pair-pulse stimulation at an interval of 100 ms from *Kcna1*^+*/*+^ (black traces) and *Kcna1*^*−/−*^ mice (red traces). In response to the paired-pulse stimuli, facilitation of synaptic events was observed in both *Kcna1*^+*/*+^ and *Kcna1*^*−/−*^ mice. Notably, the PPR obtained by two successive GABAergic eIPSCs was increased in *Kcna1*^*−/−*^ compared with *Kcna1*^+*/*+^ mice (Fig. 4e). Changes in PPR reflect alterations in release probability from the presynaptic terminals^65,66^ thus the enhanced PPR suggests that the release probability from GABAergic presynaptic terminals is decreased at mITC-CeL synapses in *Kcna1*^*−/−*^ mice. Together, the responses evoked by local stimulation of mITCs demonstrate that inhibitory synaptic transmission onto CeL neurons is significantly impaired in *Kcna1*^*−/−*^ mice.

### Feed-forward inhibition mediated by mITCs neurons onto CeL is impaired in *Kcna1*^*−/−*^ mice

**Figure 5:**
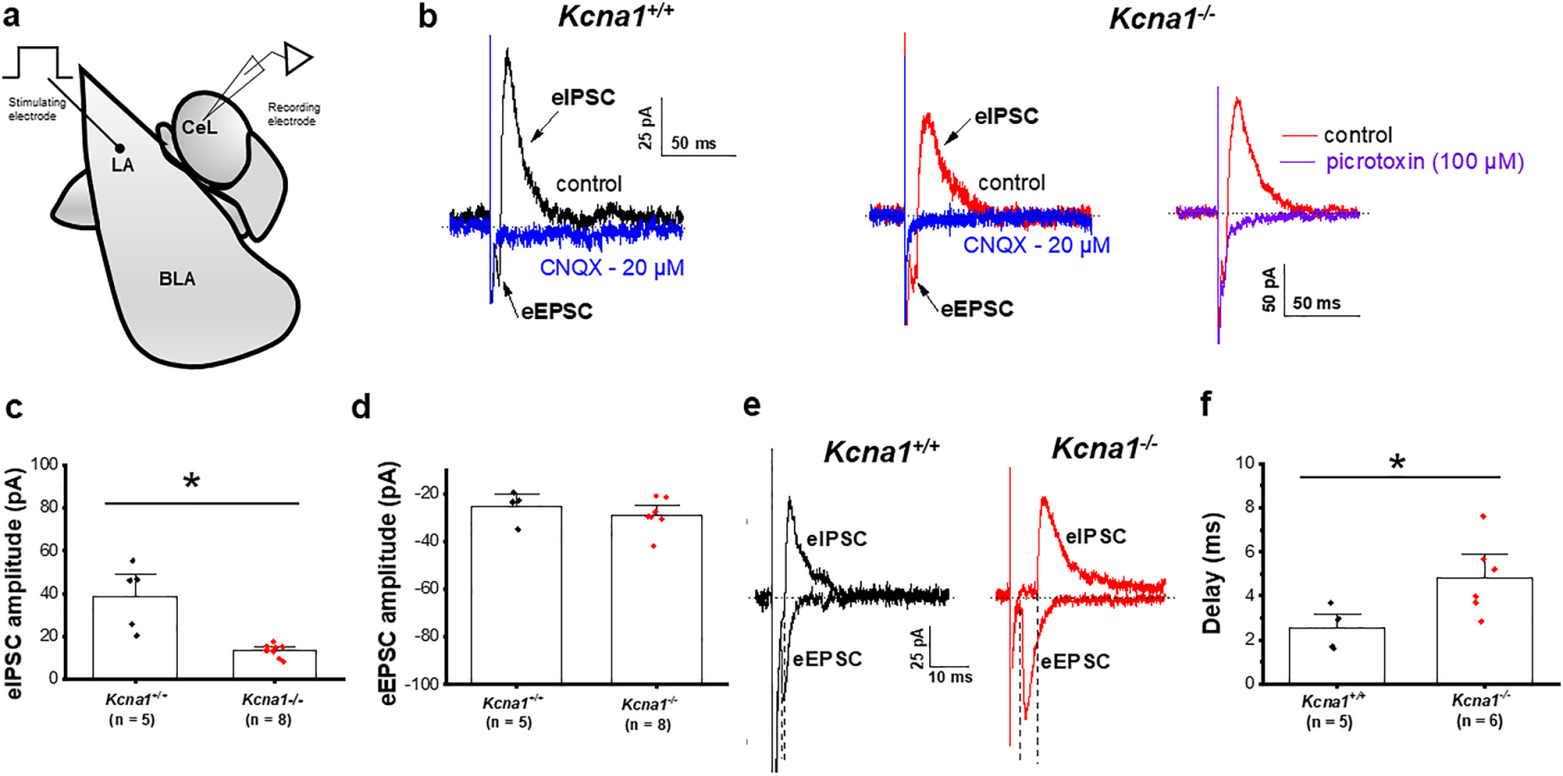
*Kcna1*^*−/−*^ mice exhibit impaired feed-forward inhibition in the LA-CeL pathway. **(a**) Schematic of a coronal section of amygdala complex showing the stimulating electrode placed in the LA to evoke synaptic responses onto CeL neurons. (**b**) Representative synaptic current traces evoked by LA stimulation were recorded from CeL interneurons in *Kcna1*^+*/*+^ (left) and *Kcna1*^*−/−*^ (right) mice at a holding potential of − 20 mV. LA stimulation resulted in a biphasic response, composed on monosynaptic glutamatergic (eEPSC) and a disynaptic (eIPSC) component. Application of CNQX (20 µM, blue trace) abolished both the eEPSC and eIPSC confirming that eIPSC component is driven by excitatory synapses on inhibitory mITC neurons projecting onto CeL neurons of both in *Kcna1*^+*/*+^ and *Kcna1*^*−/−*^ mice. In *Kcna1*^*−/−*^ mice, the outward currents was blocked by GABA_A_ receptor antagonism, picrotoxin (100 µM, violet trace), suggesting that eIPSCs were mediated by a disynaptic response. (**c**,**d**) Mean values of eIPSC **(c)** and eEPSC **(d)** amplitude. The data show that eIPSC amplitude was significantly reduced in *Kcna1*^*−/−*^ mice compared to *Kcna1*^+*/*+^ mice. No significant changes in eEPSC amplitude were observed. *Kcna1*^+*/*+^, n = 5 cells from 4 mice; *Kcna1*^*−/−*^, n = 8 cells from 5 mice. (**e**) Representative eEPSC and eIPSC currents evoked by LA stimulation were recorded from CeL interneurons in *Kcna1*^+*/*+^ (black trace) and *Kcna1*^*−/−*^ (red trace) mice at a holding potential of 0 mV (eIPSC) and − 70 mV (eEPSC). To measure the synaptic delay between the eEPSC and eIPSC components the peak of the eEPSC and eIPSC currents were measured and the 10% level was detected (dashed lines). (**f**) Mean values of the synaptic delay between the eEPSC and eIPSC component. The data show that synaptic delay was significantly longer in *Kcna1*^*−/−*^ mice compared to *Kcna1*^+*/*+^ mice. *Kcna1*^+*/*+^, n = 5 cells from 4 mice; *Kcna1*^*−/−*^, n = 6 cells from 5 mice. Together, the reduction of the eIPSC component and longer synaptic delay suggests feed-forward inhibition is impaired in CeL interneurons of *Kcna1*^*−/−*^ mice. Dotted lines indicate the base line. *LA* lateral amygdala, *BLA* basolateral amygdala, *CeL* central lateral amygdala. The schematic shown in *a* was made using curved line tool in PowerPoint. *p < 0.05.

As noted, the mITCs mediate feed-forward inhibition (FFI) onto neurons of the CeL, playing an important role in sculping amygdala network dynamics^53,62,63^. To test whether loss of K_v_1.1 subunits results in altered disynaptic FFI of CeL neurons, we applied electrical stimulation in the lateral amygdala while recording synaptic responses in CeL neurons from *Kcna1*^+*/*+^ and *Kcna1*^*−/−*^ mice (Fig. 5a). At a holding potential of – 20 mV, electrical stimulation of the lateral amygdala elicited a biphasic response in CeL neurons (Fig. 5b) that is composed of a monosynaptic excitatory (eEPSC) component and a disynaptic inhibitory (eIPSC) response. The inhibitory component was blocked by bath perfusion of the non-NMDA receptor antagonist CNQX (20 µM) (Fig. 5b, blue trace), indicating that the eIPSC component is driven by excitatory synapses onto inhibitory mITCs neurons projecting onto CeL neurons^62^. In *Kcna1*^*−/−*^ mice, bath application of the GABA_A_ receptor antagonist picrotoxin (100 µM) also completely blocked the inhibitory component (Fig. 5b, right, violet trace) confirming that disynaptic transmission is mediated by mITCs GABAergic neurons. Interestingly, the eIPSC amplitude was significantly reduced in *Kcna1*^*−/−*^ mice (16.2 ± 3.0 pA, n = 5 cells from 4 mice) compared with *Kcna1*^+*/*+^ mice (38.8 ± 6.6 pA, n = 8 cells from 5 mice; *p* = 0.004, Fig. 5c). No changes on the eEPSC component in CeL neurons were observed (Fig. 5d). The eEPSC amplitude values were − 25.2 ± 3.3 pA for *Kcna1*^+*/*+^ mice (n = 5 cells from 4 mice), and − 25.8 ± 3.6 pA for *Kcna1*^*−/−*^ mice (n = 8 cells from 5 mice), respectively. It is known that feedforward inhibitory circuits permit a narrow window between excitatory and inhibitory inputs so that disynaptic inhibition occurs after a short delay from the onset of monosynaptic eEPSCs; this synaptic delay restricts the time window for temporal summation of excitatory inputs^67^. Given the importance of FFI inhibition in regulating the timing of neuronal responses, we measured the timing of each component of FFI in CeL neurons in response to stimulation of LA by voltage clamping the membrane at their respective reversal potentials (− 70 mV for eEPSC and 0 mV for eIPSC; Fig. 5e). In both, *Kcna1*^+*/*+^ (black trace) and *Kcna1*^*−/−*^ (red trace) mice the eIPSC component occurred with a delay following the onset of eEPSC component. However, the mean delay between the eEPSC and eIPSC was found to be significantly longer in *Kcna1*^*−/−*^ mice (4.86 ± 0.6, n = 5 cells from 4 mice) compared with *Kcna1*^+*/*+^ mice (2.58 ± 0.3 ms, n = 6 cells from 5 mice, *p* = 0.026; Fig. 5f). These data suggest K_v_1.1-containing channels play an important role in regulating the spike integration window in CeL GABAergic interneurons by setting a limit for the spike generation provoked by the temporal summation of excitatory synaptic activity.

## Discussion

To our knowledge, the current study is the first to provide evidence of key roles of K_v_1.1-containing potassium channels in regulating network excitability of the amygdala circuitry. Using *Kcna1* null mice (*Kcna1*^*−/−*^), a well-known mouse model of temporal lobe epilepsy, here we show that deletion of K_v_1.1 subunits alters intrinsic excitability and synaptic activity in neurons from two amygdalar subdivisions known to be involved in epilepsy and epilepsy-related comorbidities.

At the cellular level, we found no significant differences in either passive membrane properties or action potential parameters of BLA pyramidal neurons between wild-type and *Kcna1*^*−/−*^ mice (Fig. 1). Unaltered excitability has been previously reported in hippocampal CA3 and layer V neocortical pyramidal neurons from *Kcna1*^*−/−*^ mice^21,68^. Although immunocytochemical studies have shown K_v_1.1 subunits to be localized in BLA pyramidal neurons^49^ it is likely that the lack of changes in somatic firing responses observed here could reflect the low level of K_v_1.1 channel expression in the somatic compartment. This notion is consistent with a pharmacological study of lateral amygdala pyramidal neurons in which application of dendrotoxin-K, a selective blocker of K_v_1.1-containing channels, did not modify intrinsic firing properties^69^.

Presynaptic K_v_1.1-containing channels localized in BLA neurons play a critical role in mediating the inhibitory effect of µ-opioids on neurotransmitter release from GABAergic inputs to the BLA^49^. Here, we tested whether the complete loss of K_v_1.1 subunits affected the spontaneous synaptic activity of BLA pyramidal neurons. Recordings of spontaneous postsynaptic currents provide information concerning the overall synaptic drive within an intact network, which in turn has functional implications for the overall output of a given neuron^66^. It is well established that alterations in the glutamatergic and GABAergic systems contribute to hyperexcitability in many animal models of epilepsy and other neurodevelopmental disorders including autism and schizophrenia^70^. Examining whether there exists any differences in excitatory and inhibitory spontaneous synaptic activities in the BLA of *Kcna1*^*−/−*^ mice we found that the frequency of sEPSCs was increased in BLA neurons in *Kcna1*^*−/−*^ mice (Fig. 2c). This result is in agreement with studies in animal models of epilepsy showing that alterations in BLA pyramidal neurons induced by repeated seizures or kindling are confined to the presynaptic glutamatergic terminals^45,46^. In contrast to sEPSCs, analysis of sIPSCs shows no differences in the frequency or amplitude of inhibitory GABAergic currents between *Kcna1*^*−/−*^ and *Kcna1*^+*/*+^ mice (Fig. 2e,f). We hypothesize that the noted shift in the E-I balance due to the increase in sEPSC frequency may contribute to the epileptiform activity of the *Kcna1*^*−/−*^ mice.

Loss of K_v_1.1 function is known to affect the near-threshold excitability and repetitive firing properties of fast-spiking neocortical^6,55^ and auditory GABAergic interneurons^71^. Within the amygdala complex, the lateral subdivision of central amygdala (CeL) is under strong local inhibitory control and it has been reported that the CeL mediates autonomic and behavioral responses associated with comorbidities of epilepsy such as depression, anxiety and pain via projections to the brain stem^35,36,72^. As such, alterations in the intrinsic excitability of CeL neurons can affect the functional output of the amygdala circuitry. A previous report on CeL neurons showed that α-dendrotoxin (DTX)—sensitive K_v_1-containing channels of the K_v_1 family (K_v_1.1–1.6) regulate spike latency of LS cells^58^, although effects of K_v_1.1-containing channels on CeL interneurons was not explicitly tested. In the present study, we demonstrate that the lack of K_v_1.1 subunits has a major influence on the excitability of LS cells of the CeL by reducing the characteristic delay to first AP (Fig. 3b), decreasing the rheobase (Fig. 3c), and increasing the number of APs in response to current injections (Fig. 3e). These data suggest that K_v_1-containing channels contribute to attenuating depolarizing voltage transients caused by excitatory synaptic inputs in LS cells, allowing for a delay in the response towards a sustained barrage of excitatory synaptic inputs^73–75^. Altogether, an increase in action potential firing in response to somatic stimulation in late firing CeL neurons, presumably corresponding to a subset of GABAergic neurons known to exert inhibitory control of brainstem projecting neurons of the central medial amygdala (CeM)^76,77^, would result in disinhibition of CeM output. Overall, it is likely that the enhanced excitability and AP firing of CeL LS interneurons contribute to the hyperexcitability and seizure occurrence in *Kcna1*^*−/−*^ mice.

The synaptic plasticity of the CeL is implicated in a variety of behavioral phenotypes and neurological disorders, including in models of conditioned fear and pain^78,79^. CeL interneurons, receive inputs from amygdalar and extra amygdalar sites and send GABAergic outputs to various downstream targets including the hypothalamus and brainstem. Within the amygdala complex, the CeL receives excitatory inputs from the lateral amygdala and feedforward inhibitory inputs from the GABAergic medial intercalated cells (mITCs) and send outputs to the CeM and brainstem regions such as parabrachial nucleus and nucleus tractus solitarius. Having shown that K_v_1.1-containing channels play a critical role in regulating the intrinsic excitability of CeL neurons, we also explored the potential impact of K_v_1.1 subunits on synaptic plasticity of CeL neurons. We first studied the effect of K_v_1.1 loss on the direct source of inhibitory input onto CeL neurons (Fig. 4). Recording from CeL neurons and stimulating monosynaptic inputs from local GABAergic interneurons from the mITCs (Fig. 4a), we found that in *Kcna1*^*−/−*^ mice monosynaptic IPSCs evoked at the mITC-CeL synapse had lower amplitudes than those recorded from *Kcna1*^+*/*+^ mice (Fig. 4c). This suggests that basal neurotransmission at mITC-CeL synapses are impaired in *Kcna1*^*−/−*^ mice. To explore the mechanisms underlying this synaptic impairment, we investigated the possible involvement of presynaptic mechanisms by measuring the pair-pulse ratio (PPR) of eIPSCs, a parameter affected by changes in release probability from presynaptic terminals^65^. We found that the PPR at mITC-CeL synapses was significantly increased in *Kcna1*^*−/−*^ mice compared to *Kcna1*^+*/*+^ mice, suggesting that the release probability from the GABAergic terminals is decreased in the mITC-CeL pathway in *Kcna1*^*−/−*^ mice (Fig. 4e). Enhanced short-term facilitation of mITC-evoked IPSCs indicates modification in the frequency filtering properties in the mITC-CeL pathway via rapid changes in synaptic strength^80^, allowing the sensitization towards high frequency or coincident activation of inhibitory mITC inputs onto CeL. Therefore, *Kcna1* subunits seems to be critical to maintain a precise control of temporally organized activity in the CeL. Together, these data show that K_v_1.1-containing channels are crucial contributors towards the synaptic strength and short-term plasticity of CeL neurons.

CeL neurons establish reciprocal connections whose functional role has not yet been clarified^54^, and it is difficult to exactly infer how *Kcna1*-containing channels regulate CeL overall output activity. The CeL receives glutamatergic afferents from the nociceptive parabrachial pontine nucleus of the brainstem^81^ and from the LA, where thalamic and cortical multimodal sensory inputs converge, conveying information associated to fear learning and emotional processing^82^. We speculate that different local inhibitory networks regulate distinct behavioral outcomes depending upon context and that dysregulation of amygdala circuits in *Kcna1 *^*−/−*^ mice might result in responses exacerbated or inappropriate for that context*.*

Feedforward inhibition (FFI) is an important mechanism previously characterized in hippocampal^67^ and cerebellar^83^ neurons, where it is shown to play a key role in controlling spike timing. In thalamic networks^84^ FFI restrains the propagation of epileptiform waves^85,86^. Dysfunction of FFI is known to cause abnormal circuit dynamics that underlie seizures; reduced FFI in fast-spiking interneurons has been implicated in the fast spreading of epileptic seizures in a mouse model of Dravet syndrome^87^. Here we showed the involvement of K_v_1.1-containing channels in FFI of CeL neurons in an animal model of TLE. Our results are consistent with previous reports that excitatory projections from the lateral amygdala not only project to the CeL directly but also target inhibitory interneurons in the mITC which in turn inhibit CeL neurons via a FFI mechanism^53,62,63^. The lack of K_v_1.1 subunits specifically decreased the amplitude of the inhibitory component of disynaptic responses in CeL neurons after LA stimulation (Fig. 5c). It is interesting to note that there were no changes in the amplitude of the eEPSC component (Fig. 5d), indicating that reduction of FFI onto CeL neurons must be located downstream of the excitatory input. FFI mediated by mITCs is shown to underlie fear extinction^88^ thus any reduction could induce impairment of fear extinction and/or anxiety in *Kcna1*^*−/−*^ mice.

It is well established that FFI sets a limit for overexcitation by temporally restricting the time window for temporal synaptic integration during which action potentials can be generated^67^. Our study suggests that K_v_1.1-containing channels contribute to the regulation of the spike integration window in CeL interneurons. The spike integration window in *Kcna1*^*−/−*^ mice was wider (long delay between the eEPSC and eIPSC component) compared to *Kcna1*^+*/*+^ mice (Fig. 5e,f), which could potentially allow multiple scattered inputs to trigger action potentials in CeL neurons. Fundamentally, altering the spike integration window will alter how information is processed as a function of frequency. Together, our results suggest that K_v_1.1-containing channels basally regulate disynaptic feed-forward inhibition in LA-CeL pathway. Impairment of feedforward inhibitory control of CeL outputs could affect the multisensory integration of cortical and thalamic inputs converging onto the amygdala, ultimately modifying emotional behaviors and nociceptive responses in epilepsy-related conditions. Such impairment may also compromise the function of the central autonomic network involved in cardiorespiratory regulation increasing the risk of SUDEP in *Kcna1*^*−/−*^ mice.

GABAergic neurotransmission has long been considered as a mechanism to restrain excessive excitation in neuronal networks and thus prevent the occurrence of seizures^89^. However, in experiments using human tissue from temporal lobe epilepsy patients it was shown that synchronous activity of pyramidal cells mediates transition from inter-ictal discharges to focal seizure events while interneuron firing leads to inter-ictal epileptiform discharges relying on both GABAergic and glutamatergic activity^90^. Furthermore, interneuron hyperexcitability contributes to seizure onset by promoting extracellular K^+^ accumulation, allowing the recruitment of surrounding areas and increasing the propensity to sustain synchronous seizure activity^91,92^. Our data showing increased firing activity in *Kcna1*^*−/−*^ CeL interneurons is consistent with network hyperactivity which may promote seizures through loss of spike timing control in the local circuit.

In conclusion, our results provide the first evidence concerning critical roles of K_v_1.1-containing channels in synaptic integration and shaping the properties of a feedforward inhibitory circuit within the CeL. While *Kcna1*^*−/−*^ mice exhibit spontaneous seizures, it remains to be determined how these K_v_1.1-related mechanisms directly or indirectly affect seizure initiation and propagation within the amygdala and translate into complex phenotypes characterized by comorbid behaviors and respiratory dysfunction.

## Materials and methods

### Animals

Experiments were performed on littermate wild-type (*Kcna1*^+*/*+^) and null (*Kcna1*^*−/−*^) mice. For this study a total of 29 male and 30 female mice (P22-P28) were used. Breeding pairs of heterozygous (*Kcna1*^*−/*+^*)* mice were on a C3HeB/FeJ congenic background and colonies were maintained in the Animal Resources Unit at the University of British Columbia. Mice were given food and water ad libitum and kept on a 12-h light/dark cycle. All the experimental protocols were approved by the University of British Columbia Animal Care Committee (UBC ACC Protocol A19-0233) and were in accordance with the Canadian Council on Animal Care (CCAC) guidelines. The present study complies with the pertinent aspects of ARRIVE guidelines.

### Acute brain slice preparation

Under isoflurane anesthesia [5% (vol/vol) in oxygen], mice were decapitated, and brains removed and transferred immediately to an ice-cold oxygenated (95% O_2_-5% CO_2_) sucrose cutting solution containing the following (in mM): 214 Sucrose, 26 NaHCO_3_, 1.25 NaH_2_PO_4_, 11 glucose, 2.5 KCl, 0.5 CaCl_2_, and 6 MgCl_2_ (pH 7.4). 300 µm-thick coronal slices of amygdala complex containing basolateral and central amygdala were collected using a vibratome (VT 1200; Leica). Slices were incubated in artificial cerebral spinal fluid (ACSF) containing (in mM):126 NaCl, 26 NaHCO_3_, 2.5 KCl, 1.5 NaH_2_PO_4_, 2 MgCl_2_, 2 CaCl_2_,10 glucose (pH 7.4) with 95% O_2_-5% CO_2_ at 37 °C for 45 min prior to recording.

### Whole cell patch clamp recordings

After incubation in ACSF for ~ 40 min, individual slices were transferred to the recording chamber, superfused with ACSF and maintained at 30 °C. BLA and CeL amygdala neurons were visualized with infrared differential interference contrast (IR-DIC; Slicescope 6000 Scientifica, UK) in combination with a X40 water immersion objective. Whole-cell patch-clamp recordings were performed to record intrinsic excitability and synaptic properties. All recordings were collected using a Multiclamp 700B amplifier and signals were digitized and acquired using Digidata 1550 and pClamp 10 and/or 11 software (Molecular devices). The recording chamber was grounded with an Ag/AgCl pellet. Patch pipettes (4–6 MΩ) were pulled from borosilicate glass using a P-1000 micropipette puller (Sutter Instruments).

Intrinsic excitability was recorded in current clamp mode using an internal recording solution containing the following (in mM): 120 K-gluconate, 10 HEPES, 1 MgCl_2_, 1 CaCl_2_, 11 KCl, 11 EGTA, 4 MgATP, 0.5 NaGTP, with pH adjusted to 7.2 using KOH and osmolarity adjusted to 290 mOsm/kgH_2_O using D-mannitol. The liquid junction potential was + 13.3 mV and the data was reported without subtraction. Bridge balance values were monitored during recordings and cell displaying bridge balance values > 30 MΩ were excluded from the analysis. Action potentials were evoked with 1.2 -s long square-pulse current injections from -100 to 200 pA with increments of 10 pA. Data under current clamp conditions were sampled at 50 kHz and low-pass filtered at 10 kHz.

Synaptic activity such as spontaneous and evoked postsynaptic currents were recorded in voltage clamp mode using a cesium based internal solution containing the following (in mM): 140 Cs-methanesulfonate, 5 CsCl, 5 tetraethylammonium-Cl, 1 EGTA, 10 HEPES, 4 MgATP, 0.5 NaGTP, the pH was adjusted to 7.2 with CsOH, and osmolality was adjusted to 290 mOsm/kgH_2_O with D-mannitol. To isolate spontaneous excitatory post synaptic currents (sEPSCs), the GABA_A_ receptor antagonist picrotoxin (PTX, 100 µM) was added to the ACSF. To isolate spontaneous inhibitory postsynaptic currents (sIPSCs), the NMDA receptor antagonist D-2-amino-5-phosphonovalerate (D-APV, 50 µM), and AMPA receptor antagonist cyanquixaline 6-cyano-7-nitroquinoxaline-2,3-dione (CNQX, 20 µM) were added to the ACSF. To evaluate sIPSCs, cells were held at a membrane potential of 0 mV, and for sEPSCs cells were held at a membrane potential of − 70 mV during a 60-s gap-free recording. Data acquisition was sampled at 20 kHz and low-pass filtered at 2 kHz. Recordings with a series resistance > 25 MΩ were excluded from analysis.

To evoke synaptic responses electric pulses were generated with an S48 stimulator via a stimulus isolation unit (SIU5; Grass Instruments) and applied with a concentric bipolar electrode (CBAPC100; FHC Inc.). Evoked inhibitory postsynaptic currents (eIPSCs) were elicited in CeL amygdala GABAergic interneurons when the stimulating electrode was placed on the medial intercalated cells (mITC). As shown in Fig. 4a (middle panel) anatomical landmarks were used to identify the mITC, located within the intermediate capsule.

eIPSCs were isolated in the presence of CNQX (20 µM), D-AP5 (50 µM) and GABA_B_ blocker CGP52432 (1 µM) in ACSF recording solution. For feed-forward experiments, the stimulating electrode was placed in the lateral amygdala (LA) and evoked synaptic responses were recorded in CeL neurons at a holding potential of − 20 mV. For morphologic identification of BLA and CeL neurons, recorded neurons were labeled with biocytin (0.05% in the internal solution) by applying hyperpolarizing current for 20 min in current-clamp mode. Subsequently, the recording pipette was withdrawn slowly and the slice was immediately fixed into 4% paraformaldehyde (PFA) solution at 4 °C. Biocytin-filled neurons were visualized by incubating the slices in avidin-biotinylated-horseradish peroxidase (ABC). Images of biocytin-filled neurons were obtained using an Olympus BX-53 Widefield Microscope with a 40×/NA 0.8 semi-apochromat objective in order to visualize cell morphology and confirm localization (Fig. 4a, lower panel). z-stack acquisition was performed using the Fiji Image processing software (version 1.53j).

### Data analysis and statistics

Electrophysiological recordings were analyzed using Clampfit 11 (Molecular devices); data plotting, figures generation and statistical analysis were performed using Origin 8.6 (Origin Lab). The steady state frequency of action potentials was obtained from the last 500 ms period of the depolarizing current pulses and plotted as a function of normalized current injection (*f*–I relation). Rheobase was measured as the minimum intensity of 1 s current pulse required for initiation of AP. The spike delay was measured from the start of the current injection to the start of the rising phase of first AP evoked by the rheobase. Detection and analysis of the spontaneous synaptic postsynaptic currents was performed by creating a template in Clampfit 11. For calculation of sEPSCs to sIPSCs ratio, mean values of each measure (frequency and amplitude) for each cell were calculated and expressed as a ratio. The ratios were then pooled within groups and compared the ratio of the mean values. The paired-pulse ratio (PPR) was measured by delivering two pulses with an increasing interstimulus interval, and the PPR was calculated from the amplitude of the synaptic response to the second pulse divided by the first pulse. To measure the timing of feed-forward inhibition (synaptic delay between the eEPSCs and eIPSCs), the eEPSCs and eIPSCs were separated by voltage clamping at their respective reversal potentials (eEPSCs were recorded at − 70 mV and eIPSCs at 0 mV, respectively). We used respective 10% rise time points to determine the eEPSC-eIPSC delay. All data are expressed as mean ± SEM. Statistical comparison was performed with one-way ANOVA using Tukey post hoc test. Differences with *P*-value < 0.05 were considered statistically significant. The n value represents the number of cells tested. In most experiments, only one neuron was recorded from an individual animal; the sample size indicates the number of animals used for recordings. Synaptic and action potential parameters from age-matched male and female mice were pooled together for statistical comparison.

### Pharmacological agents

D-AP5, CNQX and CGP52432 were purchased from Tocris Bioscience. Picrotoxin (PTX) was purchased from Sigma Aldrich. All drugs were prepared as stock solutions (CNQX was dissolved in DMSO and the other drugs in nanopure H_2_O) and stored at − 20 °C; working aliquots were thawed and added to ACSF. Drugs were applied by bath perfusion at a flow rate of 1 ml/min.
